# Sexually transmitted infections in HIV-infected people in Switzerland: cross-sectional study

**DOI:** 10.7717/peerj.537

**Published:** 2014-08-26

**Authors:** Katharina Sprenger, John Marc Evison, Marcel Zwahlen, Cedric M. Vogt, Maria Verena Elzi, Christoph Hauser, Hansjakob Furrer, Nicola Low, Swiss HIV Cohort Study

**Affiliations:** 1 Department of Infectious Diseases, Bern University Hospital and University of Bern, Bern, Switzerland; 2 Institute of Social and Preventive Medicine, University of Bern, Bern, Switzerland; 3 Institute for Infectious Diseases, University of Bern, Bern, Switzerland

**Keywords:** Hepatitis B, Hepatitis C, Herpes simplex type 2, HIV infection, Sexual health, Sexually transmitted infections, Sexually transmitted diseases, *Chlamydia trachomatis*, *Neisseria gonorrhoeae*, Syphilis

## Abstract

Sexually transmitted infections (STI) in HIV-infected people are of increasing concern. We estimated STI prevalence and sexual healthcare seeking behaviour in 224 sexually active HIV-infected people, including men who have sex with men (MSM, *n* = 112), heterosexual men (*n* = 65) and women (*n* = 47). Laboratory-diagnosed bacterial STI were more common in MSM (*Chlamydia trachomatis* 10.7%; 95% CI 6.2, 18.0%, lymphogranuloma venereum 0.9%; 95% CI 0.1, 6.2%, *Neisseria gonorrhoeae* 2.7%; 95% CI 0.9, 8.0%, syphilis seroconversion 5.4%; 95% CI 2.0, 11.3%) than heterosexual men (gonorrhoea 1.5%; 95% CI 0.2, 10.3%) or women (no acute infections). Combined rates of laboratory-diagnosed and self-reported bacterial STI in the year before the study were: MSM (27.7%; 95% CI 21.1, 36.7%); heterosexual men (1.5%; 95% CI 0.2, 10.3%); and women (6.4%; 95% CI 2.1, 21.0%). Antibodies to hepatitis C virus were least common in MSM. Antibodies to herpes simplex type 2 virus were least common in heterosexual men. Most MSM, but not heterosexual men or women, agreed that STI testing should be offered every year. In this study, combined rates of bacterial STI in MSM were high; a regular assessment of sexual health would allow those at risk of STI to be offered testing, treatment and partner management.

## Introduction

Sexually transmitted infections (STI) are important health risks for people living with HIV infection in the era of combination antiretroviral therapy (cART) (Fakoya et al., 2007). Surveillance reports of syphilis, gonorrhoea and chlamydia have risen during the 2000s, particularly in men who have sex with men (MSM) (Bundesamt für Gesundheit, 2013; Centers for Disease Control and Prevention, 2014). In addition, there have been outbreaks of STI which were previously rare, like lymphogranuloma venereum (LGV) (Bundesamt für Gesundheit, 2005; Bremer et al., 2006; Ward et al., 2007) and hepatitis C (Wandeler et al., 2012; Yaphe et al., 2012) in HIV positive MSM.

Regular testing for STI is recommended in some countries as part of routine follow up for all people with HIV infection (California STD Controllers Association and California Coalition of Local AIDS Directors, 2001; Fakoya et al., 2007). But the levels of STI at which regular testing in people with HIV infection is worthwhile are unclear (Rieg et al., 2008). Estimating the frequency of STI can be difficult because cohort studies are challenging (Jin et al., 2007b; Rieg et al., 2008) and cross-sectional studies miss episodes diagnosed in different settings at different times. Jin et al. (2007a) tried to overcome this challenge by combining diagnoses made at yearly study visits with self-reported diagnoses in the past year in a cohort of HIV negative MSM.

There are no guidelines for regular STI testing in HIV-infected people in Switzerland. The Swiss HIV Cohort Study (SHCS) (2010) provides regular follow up and care for about 7500 people with HIV infection in Switzerland, of whom 30% are women and 35% are assumed to have acquired HIV through sex between men. The aim of this study was to inform decisions about the need for regular STI screening in HIV-infected people in Switzerland. Specific objectives were to estimate the prevalence of bacterial and viral STI and patterns of sexual health care seeking behaviour in sexually active HIV-infected MSM, heterosexual men and women.

## Methods

We enrolled patients attending routine visits to the outpatient HIV clinic of the Bern University Hospital, Switzerland from 20.05.2009 to 28.05.2010 when a clinic physician was available to explain the study. Patients were eligible for inclusion if they were aged 18 years or older, had confirmed HIV infection, were enrolled in the SHCS, had had sexual intercourse in the last 12 months and gave written consent. The doctor administered a standardised questionnaire (in German, French or English) that asked about recent sexual partner history, previous STI and case management. All patients underwent a standard physical examination and gave a first-catch urine specimen and a blood sample. Additional specimens were taken from the throat and/or rectum from patients who reported having had receptive oral or anal sex in the last year. Whilst waiting for their blood test, patients were asked to complete a supplementary questionnaire, which they posted into a box in the clinic or returned later by mail. There were additional questions about satisfaction with management for previous STI and preferences for future sexual health services. All questionnaires were labelled with only a unique identification number to protect confidentiality. Additional epidemiological, patient history and cART data were derived from the SHCS database. The Canton of Bern Ethical Committee approved the study protocol (application number 026/09).

Specimens were tested in the laboratory of the University Institute of Infectious Diseases, according to manufacturers’ instructions. First catch urine specimens were tested by polymerase chain reaction for *Chlamydia trachomatis* (COBAS TaqMan CT Test v2.0, Roche Diagnostics, Rotkreuz, Switzerland) and *Neisseria gonorrhoeae* (*opa*-based real-time PCR assay, TIB Molbiol, Berlin, Germany, on the COBAS TaqMan Analyzer, Roche Diagnostics); tests that were positive for *C. trachomatis* were further examined and typed for LGV strains (*omp*A-based in-house assay, Institute for Medical & Molecular Diagnostics, Zürich, Switzerland). Blood was tested for herpes simplex type 2 (HSV-2) antibodies (HerpeSelect 2 ELISA IgG, Focus Diagnostics, Cypress, CA, USA). Results of the most recent serological tests for syphilis, hepatitis B and hepatitis C infections were obtained from the patient’s SHCS clinical record.

Patients with a diagnosed STI received their results by telephone from the treating physician and received antibiotic treatment, where appropriate, from the clinic. Uncomplicated chlamydia was treated with azithromycin 1 g as a single dose or doxycycline 100 mg twice daily for seven days, LGV doxycycline 100 mg twice daily for 21 days. Gonorrhoea was treated with ceftriaxone 250 mg by intramuscular injection. Partner management was discussed at the same visit and partners were contacted by the study doctor, with the agreement of the patient. Outcomes of partner notification were asked at the next follow up visit.

### Statistical analysis

Our study plan assumed that 700 HIV-infected patients would visit the outpatient clinic in one year and 50% would be enrolled. Based on published studies, if the prevalence of chlamydia or gonorrhoea was 10% (35/350), 95% confidence intervals (95% CI) would be 7.0% to 13.6% (Phipps et al., 2005; Jin et al., 2007b; Dang et al., 2009).

We conducted descriptive univariable analyses separately amongst MSM, heterosexual men and women and made statistical comparisons between groups using chi-squared tests. In all analyses heterosexual women and women who have sex with women were considered as a single group because of the small number; no woman who has sex with women was diagnosed with an STI. In each group we estimated the point prevalence of STI (with 95% CI) amongst sexually active HIV-positive people tested using the number with a positive test result at the study visit as a percentage of all participants. We used the method of Jin et al. (2007a) to estimate a one-year rate of diagnosed bacterial STI by adding study-diagnosed infections and self-reported infections in the previous year. We did not do multivariable analyses because the study was not designed to investigate risk factors for STI.

## Results

**Figure 1:**
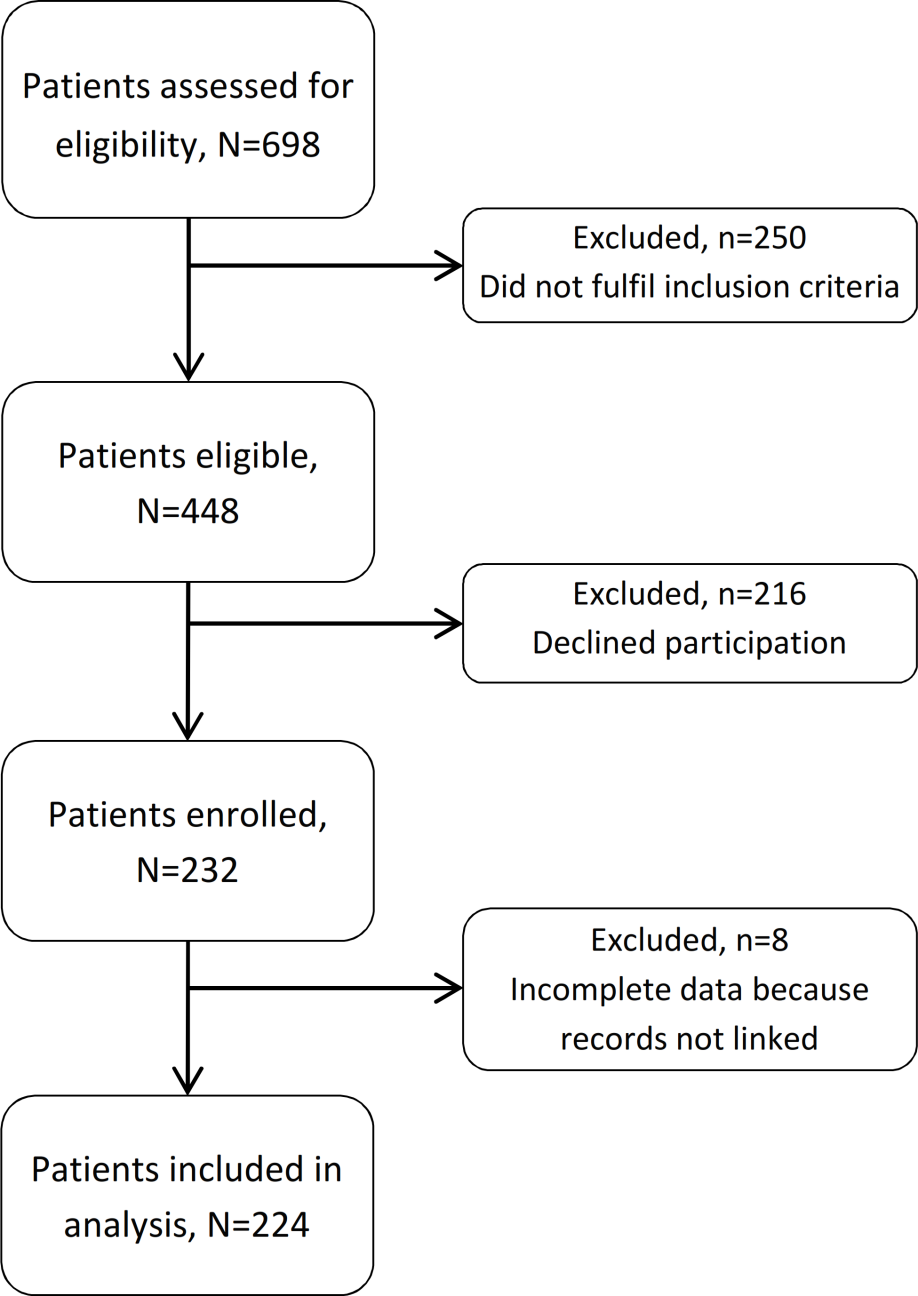
Flow chart of included and excluded patients.

We enrolled 232 HIV-infected patients during the study period. Study clinicians assessed 698 patients for eligibility (Fig. 1). Of these, 250 (36%) did not fulfil the inclusion criteria and 216 (31%) declined to participate. Amongst the enrolled patients (232/448, 52% of those eligible), eight were excluded from analysis because their records could not be linked to an SHCS number. Amongst the 224 included study participants there were 177 men (112 men who have sex with men and 65 heterosexual men) and 47 women (44 heterosexual and 3 women who have sex with women).

### Characteristics, STI Risks and symptoms amongst participants

Table 1 shows the social and demographic characteristics of study participants. Their mean age was 44 years (standard deviation, SD 9.1 years), almost all had received antiretroviral therapy and most had an undetectable viral load on the day of the study visit. Compared with all patients under active follow up in Bern (The Swiss HIV Cohort Study, 2010) patients in this study were less likely to be female (20% vs. 35%).

**Table 1:** Characteristics of study participants at enrolment (*N* = 224).

Characteristic		*N*	(%)
Sex, sexual orientation	Men who have sex with men	112	(50.0)
	Heterosexual men	65	(29.1)
	Heterosexual women	44	(19.6)
	Women who have sex with women	3	(1.3)
Route of HIV acquisition	Sex between men	108	(48.2)
	Heterosexual sex	77	(34.4)
	Injection drug use	26	(11.6)
	Other or unknown	13	(5.8)
Country of birth	Switzerland	113	(50.4)
	Other	35	(15.6)
	Missing	76	(33.9)
Age in years, mean (standard deviation)		44.4 (9.1)	na
CD4 count on day of visit, cells/µ l, mean (standard deviation)		525.9 (280)	na
Viral load <40 copies/mL	Yes, received antiretroviral therapy	154	(68.8)
	Yes, never received antiretroviral therapy	2	(0.9)
	No, ever received antiretroviral therapy	16	(7.1)
	No, never received antiretroviral therapy	37	(16.5)
	Missing	15	(6.7)

**Notes.**

na not applicable MSM men who have sex with men STI sexually transmitted infection

Reported sexual behaviours differed by sex and sexual orientation (Table 2). Almost 70% of MSM reported two or more sexual partners in the last 12 months compared with 21.5% of heterosexual men and 10.7% of women (*p* < 0.001). MSM were more likely than heterosexual men or women to report having casual sexual partners alone or both casual and regular partners (*p* < 0.001). Amongst participants reporting any casual partners, ≥50% reported consistent condom use, including three quarters of MSM who reported receptive anal sex. Consistent condom use for receptive anal sex was also reported by almost three quarters of MSM reporting any regular partners. For vaginal sex, most heterosexual men, but not women, reported consistent condom use.

**Table 2:** Sexual behaviours reported by patient to physician (*N* = 224).

	MSM		Heterosexual men		Women		*P* value
**Symptoms and sexual partnerships**	**N = 112**	**(%)**	**N = 65**	**(%)**	**N = 47**	**(%)**	
Any STI symptoms on day of visit							
Yes	7	(6.4)	3	(4.6)	1	(2.1)	0.562
No	102	(91.1)	62	(95.4)	45	(97.8)	
Not reported	3	(2.7)	0	(0)	1	(2.1)	
Total number of sexual partners in past year							
1	34	(30.4)	51	(78.5)	42	(89.4)	<0.001
2–4	23	(20.5)	11	(16.9)	3	(6.4)	
5	55	(49.1)	3	(4.6)	2	(4.3)	
**Sexual partnership type(s)**							
Casual partners only	44	(39.3)	14	(21.5)	2	(4.3)	<0.001
Both regular and casual partners	34	(30.4)	3	(4.6)	4	(8.5)	
Regular partners only	34	(30.4)	48	(73.8)	41	(87.2)	
**Sexual practices, any casual partners** ^a^	**N = 78**	**(%)**	**N = 17**	**(%)**	**N = 6**	**(%)**	
Receptive anal intercourse							
Yes, consistent condom use	46	(59.0)	0	(0)	3	(50.0)	<0.001
Yes, no consistent condom use	14	(18.0)	0	(0)	0	(0)	
No or not reported	18	(23.0)	17	(100)	3	(50.0)	
Insertive anal intercourse							
Yes, consistent condom use	37	(47.4)	1	(5.9)	0	(0)	<0.001
Yes, no consistent condom use	17	(21.8)	1	(5.9)	0	(0)	
No or not reported	24	(30.8)	15	(88.2)	6	(100)	
Vaginal intercourse							
Yes, consistent condom use	4	(5.1)	14	(82.4)	2	(33.3)	<0.001
Yes, no consistent condom use	0	(0)	2	(11.8)	1	16.7)	
No or not reported	74	(94.9)	1	(5.9)	3	(50.0)	
**Sexual practices, any regular partners** ^b^	**N = 68**	**(%)**	**N = 51**	**(%)**	**N = 45**	**(%)**	
Receptive anal intercourse							
Yes, consistent condom use	37	(54.4)	0	(0)	1	(2.2)	<0.001
Yes, no consistent condom use	14	(20.6)	0	(0)	0	(0)	
No or not reported	17	(25.0)	51	(100)	44	(97.8)	
Insertive anal intercourse							
Yes, consistent condom use	31	(45.6)	0	(0)	0	(0)	<0.001
Yes, no consistent condom use	8	(11.8)	1	(2.0)	0	(0)	
No or not reported	29	(42.7)	50	(98.0)	45	(100)	
Vaginal intercourse							
Yes, consistent condom use	3	(4.4)	36	(70.6)	24	(53.3)	<0.001
Yes, no consistent condom use	1	(1.5)	14	(27.5)	17	(37.8)	
No or not reported	64	(94.1)	1	(1.9)	4	(8.9)	

**Notes.**

MSM men who have sex with men STI sexually transmitted infection

a Number with any casual partners is sum of those with only casual and those with both casual and regular partners.

b Number with any regular partners is sum of those with only regular and those with both casual and regular partners.

### Diagnosed sexually transmitted and sexually transmissible infections

Few participants (11/224, 4.9%) reported any symptoms associated with STI on the day of study enrolment (see Table 2).

### Bacterial infections

Sixteen men had positive test results for *N. gonorrhoeae*, *C. trachomatis*, or both at the study visit. The prevalence of each STI (% and 95% CI) on the day of study enrolment is shown in Table 3. Three MSM (2.7%, 95% CI 0.9, 8.0%) had gonococcal infection (two rectal, one pharyngeal). One heterosexual man had pharyngeal gonorrhoea; he did not report any male sexual partners and there was no confirmatory specimen for culture before treatment. All 13 chlamydia-infected participants were MSM; 12 (10.7%, 95% CI 6.2, 18.0%) had *C. trachomatis* serovars D-K detected (five urethral, six rectal, one pharyngeal) and one had a rectal LGV serovar 2b. One MSM had both rectal gonorrhoea and chlamydia. There were 45 participants with positive serologic tests for syphilis with the highest estimated prevalence amongst MSM (*p* < 0.001). Amongst MSM, six (5.4%, 95% CI 2.9, 11.3%) had newly reactive serological tests for syphilis during the study period.

**Table 3:** Laboratory diagnosed sexually transmitted infections (*n* = 224). Numbers in italics show the distribution of infections by site.

Infection	MSM, *N* = *112*		Heterosexual men, *N* = *65*		Women, *N* = *47*		*P* value
	*n* ^a^	% (95% CI)	*n*	% (95% CI)	*n*	% (95% CI)	
Chlamydia, serovars D-K	12	10.7 (6.2, 18.0)	0	–	0	–	0.002
*Urethral*	*5*		*0*		*0*		
*Rectal*	*6*		*0*		*0*		
*Pharyngeal*	*1*		*0*		*0*		
LGV, rectal	1	0.9 (0.1, 6.2)	0	–	0	–	0.606
Gonorrhoea	3	2.7 (0.9, 8.0)	1	1.5 (0.2, 10.3)	0	–	0.499
*Urethral*	*0*		*0*		*0*		
*Rectal*	*2*		*0*		*0*		
*Pharyngeal*	*1*		*1*		*0*		
Syphilis^b^							
Any positive serologic test	39	34.8 (26.5, 44.2)	1	1.5 (0.2, 10.3)	5	10.6 (4.5, 23.3)	<0.001
Seroconversion during study period	6	5.4 (2.0, 11.3)	0	–	0	–	0.046
HSV-2	58	51.8 (39.1, 57.5)	23	35.4 (24.7, 47.8)	28	59.6 (45.0, 72.6)	0.028
Hepatitis C, antibody positive^c^	7	6.3 (3.0, 12.6)	21	32.3 (22.0, 44.6)	10	21.3 (11.8, 35.3)	<0.001
Hepatitis B^d^							0.117
sAg or eAg positive	2	1.8 (0.4, 6.9)	6	9.2 (4.2, 19.2)	2	4.3 (1.1, 15.6)	
Core antibody positive	46	41.1 (32.3, 50.5)	19	29.2 (19.4, 41.5)	21	44.7 (31.2, 59.1)	
Surface antibody positive, core antibody negative	27	24.1 (17.0, 33.0)	15	23.1 (14.4, 34.9)	13	27.7 (16.7, 42.1)	
Non-immune	28	25.0 (17.8, 33.9)	23	35.4 (24.7, 47.8)	10	21.3 (11.8, 35.3)	

**Notes.**

a One MSM had both chlamydia and gonorrhoea.

b Serologic tests done at baseline (Treponema pallidum haemagglutination assay, TPHA) and yearly thereafter, with follow up tests done as clinically indicated.

c No result available for 11/112 MSM, 3/65 heterosexual men, 1/47 women.

d No result available for 9/112 MSM, 2/65 heterosexual men, 1/47 women.

### Viral infections

Viral infections were diagnosed serologically (Table 3); no participant with HSV-2 antibodies reported genital ulceration on the day of the study visit. HSV-2 infection was common in all groups of participants with more than half of MSM and women, and a third of heterosexual men (*p* = 0.028), having antibodies detected. In contrast, antibodies to hepatitis C were more common in heterosexual men and in women than in MSM (*p* < 0.001). Amongst patients with no recent history of injection drug use, more heterosexual men (7/46, 15.2%) and women (6/43, 14.0%) than MSM (4/109, 3.7%) had antibodies to hepatitis C (*p* = 0.038). Amongst those who acquired HIV through injection drug use, almost all had antibodies to hepatitis C (MSM, 3/3; heterosexual men 14/19; women 4/4, *p* = 0.523). Exposure to hepatitis B virus was common in all groups. A slightly higher percentage of heterosexual men (35.4%) than MSM (25.0%) or women (21.3%) were non-immune.

### Cumulative diagnoses of acute sexually transmitted infections

Participants reported diagnoses, from a list of named STI, in the one year interval before the study. Overall, 19% (21/112) of MSM and 13% (6/47) of women reported having been diagnosed with any STI. Table 4 shows the one-year cumulative rate of bacterial STI, estimated as the sum of study diagnosed and self-reported (interval) infections as reported by Jin et al. (2007a). The one-year cumulative rate of any chlamydial infection at any site was 16.1% (95% CI 10.3, 24.1%) in MSM and 4.3% (95% CI 1.1, 15.6%) in women and of gonorrhoea the rate was 4.5% (95% CI 1.9, 10.3%) in MSM and 1.5% (0.2, 10.3%) in heterosexual men. Additional self-reported interval STI included five MSM and one woman with genital warts, two MSM and three heterosexual men with genital herpes, one woman with pubic lice and one woman with scabies.

**Table 4:** Cumulative one year rate of laboratory confirmed and self-reported sexually transmitted infection diagnoses.

	MSM, *N* = *1*12			Heterosexual men, *N* = *65*			Women, *N* = *4*7		
	Study diagnosis	Interval	Cumulative	Study diagnosis	Interval	Cumulative	Study diagnosis	Interval	Cumulative
	*n* ^a^		% (95% CI)	*n* ^a^		% (95% CI)	*n* ^a^		% (95% CI)
Chlamydia, any serovar^b^	13	5	16.1 (10.3, 24.1)	0	0	–	0	2	4.3 (1.1, 15.6)
Gonorrhoea, any site	3	2	4.5 (1.9, 10.3)	1	0	1.5 (0.2, 10.3)	0	0	–
Syphilis^c^	6	10	14.3 (8.9, 22.1)	0	0	–	0	1	2.1 (0.3, 13.8)
Total number of patients with any bacterial STI^d^	31		27.7 (21.1, 36.7)	1		1.5 (0.2, 10.3)	3		6.4 (2.1, 21.0)

**Notes.**

LGV lymphogranuloma venereum STI sexually transmitted infection

a Numbers of patients with infections diagnosed at study visit taken from Table 3.

b The question asked about chlamydia infection and did not distinguish between urogenital and LGV serovars or by clinical site of infection, so these have been grouped together.

c Study diagnoses include only patients with serological evidence of new infection. Interval diagnoses include all those who said they had been diagnosed with syphilis in the previous year. Not asked if new or old diagnosis.

d Total is the number of individual patients with either a study visit or interval diagnosis of any chlamydial infection, gonorrhoea or syphilis.

### STI testing and healthcare seeking behaviour

Amongst MSM and women, a substantial percentage reported that they had been tested for STI in the 12 months before the study visit (see Table 5). Most patients who were tested reported that this had been done at an HIV outpatient clinic. The most common alternative source of STI care was private gynaecologists for women. A majority of participants in all groups reported that they felt comfortable speaking about sexual health and STI related issues with doctors in the HIV clinic. Opinions about the role of the STI clinic in offering regular STI testing differed between groups, however. About three quarters of MSM agreed that STI testing should be offered routinely every year. A sizeable minority of heterosexual men (10/48, 21%) and women (12/39, 31%) thought that STI tests should not be offered at HIV outpatient clinics.

**Table 5:** Previous sexually transmitted infection testing and management, reported by patients (*n* = 182).

	MSM^a^ *N* = 95		Heterosexual men,^a^ *N* = 48		Women^a^, *N* = 39		*P* value
	*n*	(%)	*n*	(%)	*n*	(%)	
Have you been tested for any STI in the last 12 months?							0.015
Yes, tested positive for any STI	21	(22.1)	4	(8.3)	6	(15.4)	
Yes, tested negative for all	29	(30.5)	6	(12.5)	7	(18.0)	
No, not tested	39	(41.1)	33	(68.8)	20	(51.3)	
Not known	6	(6.3)	5	(10.4)	6	(15.4)	
Where have tests for STI been done?							0.006
HIV outpatient clinic	45	(47.4)	8	(16.7)	9	(23.1)	
STI specialist	0	(0)	1	(2.1)	0	(0)	
General practitioner	4	(4.2)	1	(2.1)	0	(0)	
Gynaecologist	1	(1.1)	0	(0)	4	(10.3)	
Not tested or not known	45	(47.4)	38	(79.2)	26	(66.7)	
Can you talk about sexual health and STI at the HIV clinic?							0.336
Yes	68	(71.6)	31	(64.6)	23	(59.0)	
No	23	(24.2)	15	(31.3)	14	(35.9)	
No response	4	(4.2)	2	(4.2)	2	(5.1)	
Should STI testing be done at the HIV outpatient clinic?							<0.001
Yes, regularly	69	(72.6)	17	(35.4)	16	(41.0)	
Yes, but only if I have symptoms	13	(13.7)	10	(20.8)	3	(7.7)	
Yes, but only if I have been at risk	5	(5.3)	9	(18.8)	7	(18.0)	
No	6	(6.3)	10	(20.8)	12	(30.8)	
No response	2	(2.1)	2	(4.2)	1	(2.6)	

**Notes.**

MSM men who have sex with men STI sexually transmitted infection

a Not all participants returned a questionnaire; no responses from 17/112 MSM, 17/65 heterosexual men, 8/47 women.

## Discussion

### Summary of main findings

In this cross-sectional study of patients attending routine HIV outpatient clinics who reported having had sexual intercourse in the past year, the prevalence of laboratory diagnosed bacterial STI was higher in MSM than heterosexual men or women. The rate of bacterial STI in MSM and women increased when self-reported infections in the year before the study visit were combined with study diagnosed infections. Antibodies to HCV were least common in MSM. Antibodies to HSV-2 were least common in heterosexual men. Most MSM, but not heterosexual men or women, agreed that STI testing should be offered every year.

### Strengths and weaknesses of study methods

Strengths of this study are that it enrolled a prospective sample of both male and female patients and used standardised methods of data collection and validated laboratory tests in a single centre. The number of patients assessed for eligibility matched the sample size calculation but the precision of STI prevalence estimates was reduced because fewer patients than planned were eligible; nearly a third of patients assessed reported that they had not had sexual intercourse in the previous year. We might have missed STI diagnoses if they were, in fact, sexually active but we think this unlikely, especially since so few STI were diagnosed in the enrolled heterosexual men and women. The number of participants enrolled was comparable to that of HIV-infected patients in other single centre studies (Bachmann et al., 2005; Stolte et al., 2006; Rieg et al., 2008) with a participation rate amongst eligible patients of about 50%. To minimise disruption to the clinic during the study period, we did not collect data about those who declined participation so our interpretation assumes that non-participation was not associated with the probability of having an STI diagnosis.

### Comparison with other studies

We did not find any other prospective studies comparing the prevalence of laboratory-detected STI in MSM, heterosexual men and women. Given the role of STI in driving HIV transmission (Cohen, 2012), the number of prospective studies examining STI in any groups of HIV-infected people in the era of cART is modest. One global review found 37 studies of any design in any population published from 2000 to 2010 (Kalichman, Pellowski & Turner, 2011). Only seven of these studies examined acute bacterial STI in prospective systematic samples of HIV-infected patients in Europe, Australia or North America (Whittington et al., 2002; Bachmann et al., 2005; Phipps et al., 2005; Stolte et al., 2006; Jin et al., 2007b; Rieg et al., 2008; Dang et al., 2009) and only one included women (Phipps et al., 2005). In MSM in our study the estimated point prevalences of chlamydia (11% any site), LGV (1%), gonorrhoea (3% any site), newly reactive syphilis serology (5%) were consistent with: another study in Switzerland (11% anorectal chlamydia serovars D-K, 1% LGV) (Dang et al., 2009); Australia (3% rectal or urethral gonorrhoea, 8% rectal or urethral chlamydia) (Jin et al., 2007b); and North America (14% gonorrhoea, chlamydia or newly reactive syphilis serology) (Rieg et al., 2008), (10% gonorrhoea or chlamydia at any site) (Whittington et al., 2002). Other recent studies have estimated a higher prevalence of rectal chlamydia (Stolte et al., 2006). Amongst women, one study in San Francisco found no chlamydia but pharyngeal gonorrhoea in 6% (3/46) women (Phipps et al., 2005). Two studies that included men did not stratify their findings by sexual orientation (Bachmann et al., 2005; Phipps et al., 2005).

The cumulative rate of STI in our study, combining new diagnoses and self-reported chlamydia, gonorrhoea and syphilis in the preceding year (28%; 21%–37%) was higher than the combined rates for HIV-negative MSM estimated by Jin et al. (2007a). The prevalence of STI has also been found to be higher in HIV-positive than HIV-negative MSM in Australia (Jin et al., 2007b), Germany (Dudareva-Vizule et al., 2013) and the USA (Whittington et al., 2002). Even if some syphilis infections reported by MSM in our study were old or treated episodes (Thurnheer et al., 2010), the combined rate in our study was consistent with the findings of a prospective study of 212 MSM in two urban clinics in the USA who were tested at baseline, six and 12 months; 28% (95% CI 22%–34%) tested positive for chlamydia or gonorrhoea at any site or had newly reactive syphilis serology (Rieg et al., 2008). Amongst women, all chlamydia infections were self-reported and the combined prevalence of 4% is compatible with that of sexually active women tested opportunistically in non-HIV clinic settings (Adams et al., 2004).

Serological evidence of HSV-2 infection was common in all groups in our study and was asymptomatic in most people, as in other studies (Romanowski et al., 2009). Hepatitis C was the only infection that was more common in heterosexual men and women than in MSM, even amongst those who reported no recent history of injection drug use. This pattern of seroprevalence has been observed before in the SHCS as a whole (Rauch et al., 2005) and probably reflects infections that resulted from past injection drug use or other parenteral exposures because heterosexual transmission of hepatitis C is uncommon (Bresters et al., 1993). The exponential increase in sexually transmitted hepatitis C infection in MSM in Switzerland (Wandeler et al., 2012) began at around the time that we conducted our study. A substantial minority of patients had no serologic immunity against hepatitis B, but we could not differentiate between people who were unvaccinated, had not seroconverted, which is common in people with HIV infection, or in whom titres had waned over time.

### Interpretation and implications for STI testing

The point prevalences of asymptomatic STI in sexually active HIV-infected MSM in our study population were similar to those in countries that recommend regular testing (California STD Controllers Association and California Coalition of Local AIDS Directors, 2001; Fakoya et al., 2007). STI testing in the routine clinic setting was feasible, most MSM in our study stated that they could discuss sexual health in the HIV outpatient clinic and most would accept regular STI testing. Sampling from the rectum and pharynx of MSM was important because more than half of the chlamydia and gonorrhoea infections were detected at these sites (Dudareva-Vizule et al., 2013). STI prevalences in HIV-infected heterosexual men and women in our study were more similar to those in HIV-uninfected people. Although these patient groups are less willing than MSM to endorse routine STI testing, a sexual health assessment offers opportunities for offering hepatitis B vaccination to non-immune individuals, early treatment for hepatitis C (Taylor et al., 2014) and to find out about the reproductive health needs of HIV-infected women (Fakoya et al., 2007).

A major reason for testing and treating STI in HIV-infected people is to reduce the transmissibility of HIV. Diagnosis and treatment of asymptomatic bacterial STI has benefits for the individual if accompanied by information about infections and their prevention for patients and their sexual partners and if partners also receive treatment to prevent re-infection. Specific information for MSM about the risks of STI if they practise serosorting might be required (Marcus, Schmidt & Hamouda, 2011). Detecting and treating asymptomatic pharyngeal and rectal gonococcal infections might help to delay the spread of antimicrobial resistance, especially if antimicrobial susceptibility testing allows the prescription of appropriate antibiotic regimens (Low et al., 2014). When assessing sexual health in the HIV clinic, patients should be asked specifically about oral and anal sex without condom use so that all potential sites of infection can be sampled.

Population level benefits are less clear. There are no randomised controlled trials of the effects on HIV transmission of diagnosing and treating STI in HIV-positive MSM but a large impact is unlikely because most new HIV infections in MSM are thought to be acquired from people who are unaware that they are HIV-infected (van Sighem et al., 2012).

In summary, guidelines about STI testing amongst HIV-infected people should be based on local knowledge about STI prevalence in women and men, and their risk of exposure. Combined prevalence rates of bacterial STI in MSM in our patient population were high and further studies should be done to assess generalizability to, and the need for guidance for, Switzerland as a whole. A regular assessment of sexual health in HIV-infected people, including a sexual history, would allow those at risk of STI to be offered testing, treatment and partner management.

## Supplemental Information

10.7717/peerj.537/supp-1 Supplemental Information 1 Raw data The workbook contains five worksheets with instructions for importing into Stata. Each worksheet contains the raw data used to generate one of the tables in the manuscript.

10.7717/peerj.537/supp-2 Supplemental Information 2 STROBE checklist

## Additional Information and Declarations

### Clinical Trial Registration

The following information was supplied regarding Clinical Trial registration: ClinicalTrials.gov: NCT00973466.
